# Is Using Closed Incision Negative Pressure Therapy in Reconstructive and Oncoplastic Breast Surgery Helpful in Reducing Skin Necrosis?

**DOI:** 10.7759/cureus.38167

**Published:** 2023-04-26

**Authors:** Zaid Al-Ishaq, Ehsanur Rahman, Fathi Salem, Saima Taj, Layth Mula-Hussain, Senthurun Mylvaganam, Raghavan Vidya, Pilar Matey, Tapan Sircar

**Affiliations:** 1 Breast Surgery, Sultan Qaboos Comprehensive Cancer Center, Muscat, OMN; 2 Breast Surgery, The Royal Wolverhampton National Health Service (NHS) Trust, Wolverhampton, GBR; 3 Radiation Oncology, Sultan Qaboos Comprehensive Cancer Center, Muscat, OMN

**Keywords:** closed incision negative pressure wound therapy, breast surgery, skin necrosis, oncoplastic breast surgery, breast reconstruction

## Abstract

Introduction

Skin necrosis is a major concern of morbidity in patients undergoing reconstructive and oncoplastic breast surgery (ROBS) as it may lead to a poor aesthetic outcome, necessitate further surgery, and delay adjuvant chemotherapy and radiotherapy if required postoperatively. Some studies have reported that closed incision negative pressure therapy (ciNPT) immediately after surgery can reduce the incidence of wound complications. Our study aimed to investigate the effect of ciNPT on skin necrosis rate after ROBS.

Methods

Our study included 82 patients in a single center who underwent 121 ROBS procedures. We used conventional dressing in 42 patients (62 procedures, group A), while we used ciNPT in 40 patients (59 procedures, group B). When ciNPT dressing was introduced in our breast unit, 40 patients with 59 ROBS procedures who had ciNPT dressing were studied prospectively. The risk factors recorded were age, body mass index (BMI), history of previous radiotherapy, history of smoking, type of incision, type of operation, breast tissue specimen weight, use of neoadjuvant chemotherapy, and implant size. Skin necrosis was classified as “minor” if it was managed conservatively with regular dressings and “major” if surgical debridement in theater and/or exchange or implant removal was necessary.

Results

The incidence of overall skin necrosis in the conventional dressing group was 17.7% (11/62), while in the ciNPT group, it was higher at 25.4% (15/59), although this was not statistically significant (p = 0.51).

ciNPT also did not show a statistically significant difference from the conventional dressing in the rate of minor necrosis (18.6% versus 11.2%, respectively; p = 0.44) and major necrosis (6.7% versus 6.4%, respectively; p = 1.00)

Conclusion

Our data has shown no superiority of ciNPT in reducing skin necrosis rate in a patient undergoing reconstructive and oncoplastic breast surgery, contrary to many other published reports. However, it may have reduced rates of other wound complications such as wound dehiscence, wound infection, and hypertrophic scar formation, which were not studied in our cohort. Further studies are needed to confirm its benefits, especially in high-risk patients.

## Introduction

Breast oncoplastic surgery is a form of breast-conserving surgery that includes oncologic resection with a partial mastectomy and ipsilateral reconstruction using volume displacement or volume replacement techniques with possible contralateral symmetry surgery when appropriate [1]. Skin necrosis is a major concern in patients who undergo reconstructive and oncoplastic breast surgery (ROBS), with reported incidence ranging from 7% to 30% [2]. Skin necrosis can delay the delivery of adjuvant chemotherapy and radiotherapy, significantly impacting the aesthetic outcome, and it can necessitate further surgery. Several risk factors have been reported to increase the incidence of skin necrosis, such as age, smoking, body mass index (BMI), prior radiotherapy, breast tissue specimen weight, use of neoadjuvant chemotherapy, and type of incision [1]. Some studies suggested a reduction in skin necrosis rate by the intraoperative diagnosis of poor vascularity of the mastectomy flap using noncontact diffuse correlation spectroscopy, fluorescein dye, Wood’s lamp imaging, and laser-assisted indocyanine green angiography. However, the routine use of these techniques is debatable [1].

The mechanisms by which the advantages of closed incision negative pressure therapy (ciNPT) are thought to be mediated include increased blood flow [3,4], reduced tension on the lateral edge of the wound with decreased risk of wound dehiscence [5,6], and less accumulation of fluid underneath the closed incision [7]. Hyldig et al. [8], in their systematic review and meta-analysis of randomized clinical trials of the use of negative pressure wound therapy (NPWT) for closed surgical incisions, found that NPWT reduced the rate of wound infection and seroma with no significant effect on wound dehiscence. However, due to the heterogeneity between the included studies, they could not conclude any general recommendation. A similar study to evaluate the prophylactic use of NPWT after a cesarean section by Yu et al. [9] found similar heterogeneity, but their result suggested a reduction in surgical site infection and overall wound complications. Strugala et al. [10] found a significant decrease in surgical site infection, wound dehiscence, and length of hospital stay in their meta-analysis study.

The use of ciNPT in patients who underwent reduction mammoplasty and expander-based breast reconstructions has shown a promising result [11-15]. However, Webster et al. [16], in their Cochrane systematic review, concluded that the results are still uncertain regarding the effectiveness of negative pressure wound therapy and that further high-quality trials are needed. Similarly, Kostaras et al. [17], in their systematic review, advised that although ciNPT might be of help in healing complicated breast incisions, more evidence is needed to support that. Our study aims to investigate if ciNPT helped reduce the incidence of skin necrosis after ROBS compared with conventional dressings.

## Materials and methods

Clinical data

This study was conducted at a single center. Retrospective data were collected for all the patients who underwent ROBS and received conventional wound dressing (group A). Once ciNPT (PICO, Smith & Nephew, London, UK) was introduced in our unit, data were prospectively collected for all patients who received ciNPT dressing following ROBS (group B). Patient demographic data, wound condition, and certain risk factors were identified from electronic records. The studied risk factors were age, BMI, history of previous radiotherapy to the breast or chest wall, history of smoking, type of incision, type of operation, use of neoadjuvant chemotherapy, breast tissue specimen weight, and implant size.

Surgical technique

Four oncoplastic breast surgeons performed the ROBS operations. Generally, subcutaneous mastectomy/therapeutic mammoplasty dissection was done using the diathermy or surgical knife. Subcutaneous mastectomy was performed either as a skin-sparing mastectomy through a transverse periareolar incision, skin-reducing mastectomy through a wise pattern incision, or nipple-sparing mastectomy through an inframammary incision or skin-reducing pattern. The type of procedure performed depended on the size and location of the tumor in the breast and the preference of the patient and surgeon. Prosthetic breast reconstruction was mainly performed as a one-stage operation, i.e., fixed-volume implant reconstruction placed either in the prepectoral space using biological mesh or submuscular space using either biological mesh or dermal sling depending on the size of the dermal sling, patient choice, and surgeon preference. Contralateral reduction mammoplasty or risk-reducing mastectomy (in some patients) was performed simultaneously. The axillary lymph node staging procedures performed were either sentinel lymph node biopsy using dual tracer (technetium 99mTc and patent blue dye) or axillary lymph node clearance depending on the preoperative axillary lymph node status. All incisions were closed with our standard technique using absorbable undyed vicryl/monocryl 3-0 sutures for the deep dermis and undyed vicryl/monocryl 4-0 sutures for subcuticular skin closure. In the conventional dressing group, Steri-Strip (3M Deutschland GmbH, Neuss, Germany) was placed over the incision, and then, a surgical Opsite (Smith & Nephew, London, UK) basic wound contact absorbent dressing was applied. All dressings were removed aseptically at seven days. In the ciNPT group, PICO dressing was applied (universally set at -80 mmHg) over the closed surgical wound immediately after the operation. The dressings were checked before the patient was discharged to ensure the negative pressure system worked. The ciNPT dressing was removed on day 7 of its application.

Patients with implant-based reconstruction had two drains, one deep and another superficial to biological mesh or dermal sling. These drains stayed until the drain output was less than 20 mL/day for two consecutive days. The patient had a prophylactic antibiotic until all drains were removed. Patients were followed up after the operation every week by the operating surgeon or the breast care nurse to assess the wound status. If clinically indicated, an ultrasound scan was performed after drain removal to check for fluid collection (seroma) around the implant.

Outcome analysis

The outcome variable included the incidence of skin necrosis in each group and the need for further surgery. The skin necrosis was classified as “minor” if it was managed conservatively with regular dressings and “major” if surgical debridement in theater and/or exchange or implant removal was necessary. The skin necrosis was identified from the clinic letters and during patient follow-up in the outpatient clinic.

Both groups were similar in terms of the presence of co-variables. Numerical variables were compared using Student’s t-test, while categorical variables were compared using chi-square tests. The incidence of skin necrosis was compared between the two groups using the chi-square method. A two-sided p-value of <0.05 was considered significant. Statistical analyses were done per breast.

## Results

Eighty-two patients (121 ROBS procedures) were studied during the study period. They were divided into two groups. Group A consisted of 42 patients with 62 ROBS procedures who had conventional dressing, while group B consisted of 40 patients with 59 ROBS procedures who had ciNPT.

Both groups were similar in age, BMI, breast tissue specimen weight, and implant size (Table 1). Similarly, there was no difference statistically between the two groups based on the type of surgery, type of incision, smoking status, laterality of procedures, previous radiotherapy, and preoperative chemotherapy (neoadjuvant chemotherapy) (Table 2).

**Table 1:** Difference between the two groups based on numerical variables SD: standard deviation, ciNPT: closed incision negative pressure therapy, BMI: body mass index

	Group A (conventional dressing) (mean ± SD)	Group B (ciNPT) (mean ± SD)	p-value
Age, year	51.65 ± 10.51	51.83 ± 9.19	0.46
BMI, kg/m^2^	27.27 ± 5.15	28.23 ± 5.30	0.63
Breast tissue specimen weight, gm	390.15 ± 260.96	472.96 ± 242.10	0.94
Implant size, cc	412.16 ± 142.41	412.61 ± 157.44	0.47

**Table 2:** Difference between the two groups based on categorical variables ciNPT: closed incision negative pressure therapy, df: degrees of freedom, BL: bilateral, CL: contralateral, UL: unilateral

		Group A (conventional dressing)	Group B (ciNPT)	Chi-square	df	p-value
Incision type	Transverse	17	22	1.34	1	0.24
	Wise pattern	45	37			
Surgical procedure	Mastectomy and implant insertion	37	37	0.80	2	0.66
	Oncoplastic reduction	18	13			
	Change of implant	8	9			
Smoker	Yes	7	4	4.25	2	0.11
	No	34	43			
	Ex-smoker	21	12			
Laterality	BL	20	20	0.68	2	0.79
	CL	20	20			
	UL	22	19			
Radiotherapy	Yes	5	8	0.95	1	0.32
	No	57	51			
Neoadjuvant chemotherapy	Yes	10	12	0.36	1	0.54
	No	52	47			

The incidence of overall skin necrosis in the conventional dressing group was 17.7% (11/62), while in the ciNPT group, it was higher at 25.4% (15/59), although this was not statistically significant (p = 0.51) (Table 3).

**Table 3:** Overall, minor, and major necrosis rates for the conventional and ciNPT groups ciNPT: closed incision negative pressure therapy, ROBS: reconstructive and oncoplastic breast surgery

	Group A (conventional dressing) (%)	Group B (ciNPT) (%)	p-value
Number of ROBS	62	59	
Overall necrosis	11 (17.7)	15 (25.4)	0.51
Major	4 (6.45)	4 (6.77)	1.00
Minor	7 (11.29)	11 (18.64)	0.44

The ciNPT also did not show a statistically significant difference from the conventional dressing in the rate of minor necrosis (18.6% versus 11.2%, respectively; p = 0.44) and major necrosis (6.7% versus 6.4%, respectively; p = 1.00) (Table 3).

## Discussion

The surgical treatment of breast cancer has significantly improved with the introduction of oncoplastic procedures, increasing the variety of surgical procedures offered to patients with breast cancer [17,18]. An increasing number of breast cancer patients choose to have a skin-sparing mastectomy and immediate implant-based reconstruction [19]. While these complex surgical procedures have improved the patient’s quality of life [20], it is associated with possible postoperative complications. Skin necrosis is one of the major complications following oncoplastic and reconstructive breast surgeries. It can lead to a poor aesthetic outcome, increased cost of treatment, and, more importantly, potential delay in adjuvant therapies in some cases [2].

At our center, the necrosis rate with conventional dressing was 17.7% (minor necrosis 11.3% and major necrosis 6.4%); hence, the idea of this study arose to determine whether the use of ciNPT would decrease the incidence of skin necrosis compared to the conventional dressing. The two groups did not exhibit significant differences with respect to age, BMI, breast tissue specimen weight, and implant size, and there was statistically no difference between the two groups based on the type of surgery, type of incision, smoking status, laterality of procedures, previous radiotherapy, and preoperative chemotherapy. With the use of ciNPT, the incidence of skin necrosis was 25.4% (minor necrosis 18.6% and major necrosis 6.8%), which was higher than the conventional dressing group, but not statistically significant (p = 0.51).

Although the overall incidence of skin necrosis was high in our cohort, this is comparable to published literature [2,15,21-23]. Notably, the difference was only minor skin necrosis (11.3% in the conventional group and 18.6% in the ciNPT group). The incidence of major skin necrosis was similar in the conventional dressing and ciNPT groups (6.4% and 6.7%, respectively; p = 1.00). The use of ciNPT did not reduce the incidence of either minor or major skin necrosis (Table 3). This explains that the incidence of skin necrosis following ROBS depends not only on a multitude of factors such as age, smoking, BMI, prior radiotherapy, diabetes, breast tissue specimen weight, use of neoadjuvant chemotherapy, and type of incision but also other factors such as skin quality, thickness of the flap, and vascularity of the flap, which may not be compensated by ciNPT dressing [2].

A ciNPT system that delivers -80 mmHg or -125 mmHg has been studied in patients undergoing reduction or therapeutic mammoplasties and two stages of expander-based breast reconstruction with positive results. In patients with bilateral reduction mammoplasty, a multicenter randomized control trial by Galiano et al. [11] on 200 patients showed that ciNPT dressing (PICO®) is effective in reducing wound healing complications and dehiscence. In a similar prospective randomized study, Tanaydin et al. [12] reported significantly lower wound complications and a significant improvement in the quality of scaring in favor of the ciNPT-treated sites. Holt et al. [13] published a case series of 24 consecutive patients. Of these patients, 21 underwent therapeutic mammoplasty, while three had wise pattern skin-sparing mastectomies and immediate implant or inferior dermal flap technique. They reported a lower incidence of wound breakdown (4.2%) on the side treated with PICO (therapeutic side) in comparison to the 16.7% rate of wound breakdown on the side treated with conventional dressing (reduction side). In our study, mastectomy and fixed-volume implant reconstruction constituted the majority, 58% in the conventional and 62% in the ciNPT group. The difference between our results and the above studies could be related to the extensive dissection and thinner skin flap required in skin-sparing mastectomy compared to reduction mammoplasty and the possible tension effect on the skin from the fixed-volume breast implant [24]. These factors might explain the slightly higher skin necrosis rate in our study, most of which were minor skin necrosis (Table 3).

In a patient who had two-stage expander-based reconstruction, Gabriel et al. [14], in their retrospective study comparing postoperative breast reconstruction outcomes between patients who had closed incision negative pressure therapy (ciNPT) at -125 mmHg and those who had the standard of care (SOC) following immediate or delayed expander-implant reconstruction, reported a decrease in the incidence of skin necrosis in the ciNPT group (5.1% in the ciNPT group versus 9.3% in the SOC group; p = 0.0070). A similar result has been published by Kim et al. [15] in their retrospective study to evaluate the use of ciNPT at -125 mmHg in patients who underwent a mastectomy and expander-based reconstruction, and they found that the ciNPT group had a lower overall mastectomy flap necrosis rate (8.9% versus 23.5%; p = 0.030) and major mastectomy flap necrosis (2.2% versus 13.7%; p = 0.031) compared with the conventional dressing group. In the previous two studies, they used a two-stage expander-based breast reconstruction, while most of our patients had fixed-volume implant-based reconstruction (36/37 and 35/37 implant-based reconstruction procedures in conventional dressing and ciNPT groups, respectively), which means less tissue tension on the skin flap in the immediate postoperative period of the patient who had two-stage expander-based breast reconstruction, which can contribute to their less low necrosis rate compared to our cohort [25]. Furthermore, the weight of the breast tissue removed in our study is larger than the study of Kim et al. (470.17 ± 243.15 versus 319 ± 173.8, respectively), meaning larger skin flap surface area, which can cause less blood flow to the distal skin edges [26-28].

Despite the studies mentioned above, in a Cochrane systematic review of the use of negative pressure wound therapy for surgical wound healing by primary closure, Webster et al. [16] concluded that uncertainty remains about whether ciNPT versus standard dressing decreases or increases the incidence of mortality, dehiscence, and seroma or if it increases costs. They advocate the need for suitably powered high-quality trials. However, systematic reviews of the available evidence for the effectiveness of ciNPT in the healing of breast incisions concluded that although ciNPT might be of use in helping the healing of complicated breast incisions and the potential for better cost-effectiveness and cosmetic outcome in comparison to the standard of care dressing, larger studies were needed before reaching that conclusion [17,29].

In the current study, we minimized the confounding effects. There was no statistically significant difference in the categorical and the numerical variables between the conventional dressing group and the ciNPT group. However, our study has certain limitations. It was an observational study. It lacked randomization. Although we have controlled for comorbidities in the two groups, they are still heterogeneous patients with different healing processes. The conventional dressing group was studied retrospectively based on clinical notes of surgeons and breast care nurses. In addition, we did not include other risk factors for mastectomy flap necrosis, including the severity of ptosis and skin flap thickness or vascularity. Furthermore, our study was limited to only investigating the effect of ciNPT on the skin necrosis rate. We did not study the incidence of wound dehiscence, wound infection, or the outcome of scar healing. A randomized study may be helpful to investigate if ciNPT does reduce the incidence of skin necrosis in patients who underwent ROBS.

## Conclusions

The role of ciNPT in patients with ROBS is controversial. Our data showed no superiority of ciNPT in reducing skin necrosis rate in patients undergoing reconstructive and oncoplastic breast surgeries. However, it may have reduced rates of other wound complications such as wound dehiscence, wound infection, and hypertrophic scar formation, which were not studied in our cohort. Further studies are needed to confirm its benefits, especially in high-risk patients.

## References

[1] Chatterjee A, Gass J, Patel K (2019). A consensus definition and classification system of oncoplastic surgery developed by the American Society of Breast Surgeons. Ann Surg Oncol.

[2] Robertson SA, Jeevaratnam JA, Agrawal A, Cutress RI (2017). Mastectomy skin flap necrosis: challenges and solutions. Breast Cancer (Dove Med Press).

[3] Kanuri A, Liu AS, Guo L (2014). Whom should we SPY? A cost analysis of laser-assisted indocyanine green angiography in prevention of mastectomy skin flap necrosis during prosthesis-based breast reconstruction. Plast Reconstr Surg.

[4] Atkins BZ, Wooten MK, Kistler J, Hurley K, Hughes GC, Wolfe WG (2009). Does negative pressure wound therapy have a role in preventing poststernotomy wound complications?. Surg Innov.

[5] Malmsjö M, Huddleston E, Martin R (2014). Biological effects of a disposable, canisterless negative pressure wound therapy system. Eplasty.

[6] Wilkes RP, Kilpad DV, Zhao Y, Kazala R, McNulty A (2012). Closed incision management with negative pressure wound therapy (CIM): biomechanics. Surg Innov.

[7] Kilpadi DV, Cunningham MR (2011). Evaluation of closed incision management with negative pressure wound therapy (CIM): hematoma/seroma and involvement of the lymphatic system. Wound Repair Regen.

[8] Hyldig N, Birke-Sorensen H, Kruse M (2016). Meta-analysis of negative-pressure wound therapy for closed surgical incisions. Br J Surg.

[9] Yu L, Kronen RJ, Simon LE, Stoll CR, Colditz GA, Tuuli MG (2018). Prophylactic negative-pressure wound therapy after cesarean is associated with reduced risk of surgical site infection: a systematic review and meta-analysis. Am J Obstet Gynecol.

[10] Strugala V, Martin R (2017). Meta-analysis of comparative trials evaluating a prophylactic single-use negative pressure wound therapy system for the prevention of surgical site complications. Surg Infect (Larchmt).

[11] Galiano RD, Hudson D, Shin J (2018). Incisional negative pressure wound therapy for prevention of wound healing complications following reduction mammaplasty. Plast Reconstr Surg Glob Open.

[12] Tanaydin V, Beugels J, Andriessen A, Sawor JH, van der Hulst RR (2018). Randomized controlled study comparing disposable negative-pressure wound therapy with standard care in bilateral breast reduction mammoplasty evaluating surgical site complications and scar quality. Aesthetic Plast Surg.

[13] Holt R, Murphy J (2015). PICO™ incision closure in oncoplastic breast surgery: a case series. Br J Hosp Med (Lond).

[14] Gabriel A, Sigalove S, Sigalove N, Storm-Dickerson T, Rice J, Maxwell P, Griffin L (2018). The impact of closed incision negative pressure therapy on postoperative breast reconstruction outcomes. Plast Reconstr Surg Glob Open.

[15] Kim DY, Park SJ, Bang SI, Mun GH, Pyon JK (2016). Does the use of incisional negative-pressure wound therapy prevent mastectomy flap necrosis in immediate expander-based breast reconstruction?. Plast Reconstr Surg.

[16] Webster J, Scuffham P, Sherriff KL, Stankiewicz M, Chaboyer WP (2012). Negative pressure wound therapy for skin grafts and surgical wounds healing by primary intention. Cochrane Database Syst Rev.

[17] Kostaras EK, Tansarli GS, Falagas ME (2014). Use of negative-pressure wound therapy in breast tissues: evaluation of the literature. Surg Infect (Larchmt).

[18] McCulley SJ, Macmillan RD (2005). Planning and use of therapeutic mammoplasty--Nottingham approach. Br J Plast Surg.

[19] Dietz J, Lundgren P, Veeramani A (2012). Autologous inferior dermal sling (autoderm) with concomitant skin-envelope reduction mastectomy: an excellent surgical choice for women with macromastia and clinically significant ptosis. Ann Surg Oncol.

[20] Albornoz CR, Bach PB, Mehrara BJ (2013). A paradigm shift in U.S. breast reconstruction: increasing implant rates. Plast Reconstr Surg.

[21] Lee TJ, Oh TS, Kim EK (2016). Risk factors of mastectomy skin flap necrosis in immediate breast reconstruction using low abdominal flaps. J Plast Surg Hand Surg.

[22] Nykiel M, Sayid Z, Wong R, Lee GK (2014). Management of mastectomy skin flap necrosis in autologous breast reconstruction. Ann Plast Surg.

[23] Sue GR, Lee GK (2018). Mastectomy skin necrosis after breast reconstruction: a comparative analysis between autologous reconstruction and implant-based reconstruction. Ann Plast Surg.

[24] Lee JH, Chang CH, Park CH, Kim JK (2014). Methylene blue dye-induced skin necrosis in immediate breast reconstruction: evaluation and management. Arch Plast Surg.

[25] Sue GR, Long C, Lee GK (2017). Management of mastectomy skin necrosis in implant based breast reconstruction. Ann Plast Surg.

[26] de Vita R, Buccheri EM (2018). Nipple sparing mastectomy and direct to implant breast reconstruction, validation of the safe procedure through the use of laser assisted indocyanine green fluorescent angiography. Gland Surg.

[27] Suga H, Shiraishi T, Tsuji N, Takushima A (2017). Risk factors for complications in expander-based breast reconstruction: multivariate analysis in Asian patients. Plast Reconstr Surg Glob Open.

[28] Chirappapha P, Petit JY, Rietjens M (2014). Nipple sparing mastectomy: does breast morphological factor related to necrotic complications?. Plast Reconstr Surg Glob Open.

[29] Liew AN, Lim KY, Khoo JF (2022). Closed incision negative pressure therapy vs standard of care dressing in breast surgery: a systematic review. Cureus.

